# Prevalence of pulmonary tuberculosis in homeless individuals in the Addis Ababa City, Ethiopia

**DOI:** 10.3389/fpubh.2023.1128525

**Published:** 2023-04-06

**Authors:** Tsegaye Shamebo, Sindew Mekesha, Muluwork Getahun, Balako Gumi, Beyene Petros, Gobena Ameni

**Affiliations:** ^1^Department of Microbial, Cellular and Molecular Biology, College of Natural and Computational Sciences, Addis Ababa University, Addis Ababa, Ethiopia; ^2^Ethiopian National Tuberculosis Reference Laboratory, Ethiopian Public Health Institute, Addis Ababa, Ethiopia; ^3^Aklilu Lemma Institute of Pathobiology, Addis Ababa University, Addis Ababa, Ethiopia; ^4^Department of Veterinary Medicine, College of Agriculture and Veterinary Medicine, United Arab Emirates University, Al Ain, United Arab Emirates

**Keywords:** Addis Ababa City, homeless individuals, prevalence, pulmonary tuberculosis, risk factor

## Abstract

**Background:**

Homeless individuals are at a high risk of infection with Mycobacterium tuberculosis *(M. tuberculosis)* as compared to the general population. The number of homeless individuals has been increasing in Addis Ababa City during the last three decades due to the migration of rural inhabitants to the City for better living conditions. The objective of this study was to estimate the prevalence of pulmonary tuberculosis (PTB) and evaluate associated risk factors in homeless individuals in Addis Ababa City.

**Methods:**

A total of 5,600 homeless individuals were screened for PTB symptoms using WHO guideline between February 2019 and December 2020. Sputum samples were cultured from individuals with symptoms of PTB for mycobacterial isolation. Logistic regression analysis was used to identify factors associated with PTB.

**Results:**

The prevalence of bacteriologically confirmed cases was 1.1% (59/5,600) or 10.54 per 1000 population. Multinomial logistic regression analysis showed that being homeless for more than 5 years, body mass index (BMI) < 18.5, smoking cigarette, living in a group of more than five individuals, close contact with chronic coughers, imprisonment and HIV infection were significantly associated with the prevalence of PTB in homeless individuals (*P* < 0.05).

**Conclusion:**

In conclusion, the result of this study indicated that the prevalence of PTB in homeless individuals was higher than the prevalence of PTB in the general population of Addis Ababa City requiring for the inclusion of the homeless individuals in the TB control program.

## Background

Globally, tuberculosis (TB) is the 13th leading cause of death and the second leading infectious killer after COVID-19 (above HIV/AIDS) (1). Despite several efforts have been made in the fight against TB in the past decade, the COVID-19 pandemic overwhelmed health care system has affected TB case notification and treatment outcomes, setting back the achievements made in the fight against TB (2). In 2021, an estimated 10.6 million people fell ill with TB and 1.6 million died from the disease worldwide, an increase of 4.5% from 10.1 million TB cases in 2020. Similarly, the TB incidence rate increased by 3.6% between 2020 and 2021 because of the reduced attention given to TB control as the health sectors were overwhelmed with COVID-19 pandemic (3). The thirty high-burden TB countries accounted for 86% of new TB cases. Over 95% of reported TB cases and deaths are in low-income countries (3).

Ethiopia is one of the 30 countries with a high burden of TB, MDR-TB and TB-HIV co-infection in the world, and in 2021, the incidence of TB was estimate at 119 cases per 100,000 population in Ethiopia (3). Despite extensive global efforts to control TB, the disease is still largely affecting socially marginalized segment of the population, such as the homeless and those in other high-risk settings (4).

Homeless individuals are at a high risk of infection with *M. tuberculosis* and develop TB compared to the general population. This is not only due to their low-income status but to several risk factors like poverty, overcrowding, malnutrition, HIV infection, smoking, alcoholism, and drug abuse (5). Evidences indicated that TB is the third major cause of illness in homeless individuals, and it is estimated that homeless individuals are 10 to 85 times higher rate risk of developing latent or active TB infection as compared to the general population (6–8). The incidence of smear-positive PTB in homeless individuals in the cities of northern Ethiopian was 2.6% (9). Thus, if the TB control programs do not consider homeless individuals, the incidence of TB in this group will keep on increasing thereby posing a high risk to the general population.

The number of homeless individuals has been increasing in the Addis Ababa City during the last three decades due to the migration of rural inhabitants to the City in search of better living conditions (10). This increase in the number of homeless individuals in Addis Ababa has led to crowded living environments on the streets, which facilitates TB transmission. However, there is limited data on the TB situation in homeless individuals in Ethiopia including in the capital of the country. Therefore, this study conducted to estimate the prevalence of PTB and evaluate associated risk factors in homeless individuals in Addis Ababa, Ethiopia.

## Materials and methods

### Study setting

A cross-sectional study was conducted between February 2019 and December 2020 in Addis Ababa City, Ethiopia. Addis Ababa is the capital City of Ethiopia. Presently, the size of the its population is estimated to be 3,384,569, with an annual growth rate of 3.8% (11). The City is inhabited by diversified Ethiopian ethnic groups, and only 1% of the population of the City are foreigners. The City is fastest growing City in Africa (12) and constitutes 30% urban population of Ethiopia (11). It has the highest rural-urban migration that accounts for 40% of its population growth. Subsequently, there is an increase in the number of homeless individuals in the City. In order to support the number of homeless individuals, the City Administration established temporary shelters for homeless individuals while this study was being undertaken. Six temporary shelters were established in six sub cities including in Arada, Yeka, Bole, Akaki-Kality, Chirkos and Addis Ketema. These temporary shelters were used for the recruitment of the study participants.

### Study participant enrollment

According to the European Typology of Homelessness and Housing Exclusion (ETHOS) (13), homeless are not a separate species of people, but they are by-products of the society on the margins of which they live. These people have no home or shelter to reside in and instead reside on the corners of streets, in parks, or in public places, if not provided a residence by the governmental or non-governmental organizations (14). Homeless individuals who registered for shelter accommodation in Addis Ababa City during the study period, had a history of having been homeless for at least 1 month prior to the study period (15), age 18 years and older, and gave their written informed consent were included in the study.

These homeless individuals were screened for PTB using the WHO guideline TB symptom screening standard document (16) between February 2019 and December 2020 by trained health professionals. The symptoms include cough ≥ 2 weeks, fever ≥ 2 weeks, night sweats ≥ 2 weeks, appetite loss ≥ 2 weeks, chest pain ≥ 2 weeks and weight loss ≥ 2 weeks. Each participant completed a questionnaire with the help of trained health professionals. The questionnaire included demographic data (age, gender, total duration of homelessness, previous residence, educational background, and marital status), TB risk behaviors (cigarette smoking, alcohol consumption, chewing khat [a plant contain amphetamine-like stimulants that has legally been used for centuries in East Africa and the Southern Arabian Peninsula. Fresh leaves of this plant contain more than 40 types of alkaloid compounds, of which cathinone and cathine are known stimulants. The stimulants cause excitement, loss of appetite and immune modulation on the users (17), drug abuse, HIV status, and malnutrition] and TB specific information (history and symptoms of TB, imprisonment, history of contact with chronically coughing individuals and history of contact with TB patients) (18). All symptoms were recorded as reported by the participants and assessed by another qualified health professional.

### Case definitions

Presumptive TB cases: patients with cough ≥2 weeks, fever ≥ 2 weeks, night sweats, hemoptysis or weight loss (19). Pulmonary tuberculosis (PTB): a participant with pulmonary TB confirmed by Xpert, smear microscopy or clinically diagnosed according to the national (19) and WHO (20) TB guidelines. Bacteriologically confirmed TB case: a patient from whom at least one sputum was positive for *M. tuberculosis* by GeneXpert, smear microscopy or culture (19, 20). Clinically diagnosed TB case: a participant who did not meet the criteria for a bacteriologically confirmed case, but was diagnosed with TB by an experienced clinician and received a full course of TB treatment (19, 20).

### Sputum sample collection

A total of 5,600 homeless individuals were screened for PTB symptoms using WHO TB symptoms screening guidelines (21). Of these, 641 were screening positive and provided sputum samples for bacteriological analysis. The participants were properly advised by trained health worker on how to produce a good sputum sample. Two sputum samples were collected from each participant (21). The first sample was collected on the spot and sent to the health facilities for Gene-Xpert MTB/RIF assay [a fully automated molecular diagnostic test for TB that simultaneously detects MTBC and resistance to rifampin (RIF) in < 2 h] (22), while the second sample was collected the next day in the early morning (morning sputum) and transported to the Aklilu Lemma Institute of Pathobiology (ALIPB) TB Laboratory, Addis Ababa University in a cold box and stored at 2–8°C until processed for mycobacteria isolation as previously described (23).

### Mycobacterial isolation and identification

Sputum samples were digested and decontaminated by the modified Petroff method (24). The digested sputum samples were inoculated onto solid Lowenstein Jensen (LJ) medium. Bacterial growth was checked for contamination and fast growers in the first week. Contaminated cultures were recorded as contaminated. The cultures were observed on weekly basis and if there was no growth at the end of 8 weeks, the result was considered negative (25). The sputum samples with growth of *M. tuberculosis* were confirmed based on microscopic examination of acid-fast bacilli in a culture smear (26).

### Anthropometric measurements

The weight and height of each study participant were measured by trained health professionals in order to estimate body mass index (BMI) of the study participants. A digital scale was used to measure the weight of each study participant, and weight was measured to the nearest 0.1 kg, while height was measured with the vertical measuring rod to the nearest 0.1 cm (27, 28).

### Rapid HIV test

The HIV status of study participants was determined through the provision of pre-test counseling by trained health professionals. Briefly, a whole blood sample was collected by finger prick. The presence of antibodies against HIV-1 and HIV-2 was determined by using HIV antibody colloidal gold (1 + 2) rapid diagnostic kits (KHB, Shanghi Kehua Bio-engineering Co Ltd, China) as a screening test, followed by HIV$\frac{1}{2}$ STAT -PAK^®^ (Chembio Diagnostics, USA), when the KBH result was reactive. When the STAT-PAK^®^ result was discordant with KBH, a third test, Unigold TM HIV (Trinity Biotech, Ireland), was also used as a tiebreaker to determine the test result following the manufacturer's instructions. Finally, post-test counseling was provided to all participants (29).

### Quality control

The questionnaire was prepared in English and translated into the local language (Amharic) and then back-translated into English by an expert fluent in both languages to maintain consistency. The questionnaire was pretested among 30 (5%) randomly selected homeless individuals before the actual data collection. The project supervisor and principal investigator strictly followed the data collection process day to day. Reagents and culture media were checked for sterility and performance characteristics in each batch of newly prepared reagent lots.

### Data analysis

The data captured in the questionnaires were pooled, error-checked, cleaned, and entered into Microsoft Excel version 2013 for storage. Later the data were transferred to SPSS version 26 statistical software and analyzed. Descriptive statistics were used to summarize socio-demographic, behavioral, environmental factors and the morbidity history of the patients. BMI was used to assess nutritional status. BMI was calculated as = weight (in kilograms)/height (in meters). A BMI value of < 18.5 kg/m^2^ was considered underweight (27). Cross-tabulation was performed between the potential risk factors and prevalence of PTB for the determination of chi-square and *p*-values. Binomial and multinomial logistic regression analyses were performed to identify potential risk factors associated with PTB. In the multivariable logistic regression analysis, adjusted Odds Ratio (AOR) and 95% confidence interval (CI) were determined. *P*-value < 0.05 was considered statistically significant.

### Ethical consideration

Ethical clearance prior to data collection was obtained from the Addis Ababa University, College of Natural and Computational Sciences Institutional Review Board (IRB) and Addis Ababa City Administration Health Bureau. Then, an official permission letter was obtained from Addis Ababa City Administration, Labor and Social Affairs Bureau. Written informed consent was obtained from all study participants after providing adequate information on the possible benefits and risks of the study in the local language (Amharic). Those participants who tested positive for TB and/or HIV infection were connected to health facilities in temporary shelters for treatment and follow-up. It is noteworthy that the decision to decline screening did not affect shelter access. Patient disease status was kept confidential through the use of anonymous personal identifiers.

## Results

### Socio-demographic characteristics of the study participants

The sociodemographic characteristics of the study participants is presented in Table 1. Majority of the participants (80.3%) were male and their mean ± SD age was 27.8 ± 9.5 years. Significant proportion of the participants addicted to smoking (82.1%) and alcohol (78.0%). Addictions to khat and drug were observed only in 1.1% and 1.0% (31/2,980) culture positive PTB cases.

**Table 1:** Socio-demographic characteristics of homeless individuals (*n* = 5, 600) with bacteriological confirmed PTB prevalence, Addis Ababa, Ethiopia, 2021.

**Variable/characteristics**		**Total *n* (%)**	**TB culture positive *n* (%)**	**TB culture negative *n* (%)**	***P*-value**
Gender	Female	1,100 (19.6)	7 (0.6)	1,093 (99.4)	0.372
	Male	4,500 (80.4)	52 (1.2)	4,448 (98.8)	
Age	18–27	753 (13.4)	6 (0.8)	747 (99.2)	0.820
	28–37	2,744 (49.0)	34 (1.2)	2,710 (98.8)	
	38–47	1,540 (27.5)	15 (1.0)	1,525 (99.0)	
	48–57	372 (6.6)	3 (0.8)	369 (99.2)	
	58 and older	191 (3.4)	1 (0.5)	19099.5	
Marital status	Single	4,869 (86.9)	49 (1.0)	4,820 (99.0)	0.652
	Married	158 (2.8)	6 (3.8)	152 (96.2)	
	Divorced	374 (6.7)	3 (0.8)	371 (99.2)	
	Widowed	199 (3.6)	1 (0.5)	198 (99.5)	
Educational status	Illiterate	2,230 (39.8)	30 (1.3)	2, 200 (98.7)	0.332
	Primary school	2,632 (47.0)	23 (0.9)	2,610 (99.1)	
	Secondary school	704 (12.6)	6 (0.8)	698 (99.2)	
	Higher education	35 (0.6)	0 (0)	35 (0.6)	
Former residence	Urban	920 (16.04)	18 (2.0)	902 (98.0)	0.328
	Rural	4,680 (83.6)	41 (0.9)	4,639 (90.1)	
Previous region	Addis Ababa	126 (2.3)	2 (1.6)	124 (98.4)	0.858
	Amhara	1,329 (23.7)	14 (1.1)	1,315 (98.9)	
	Tigray	11 (0.2)	(0)	11 (0.2)	
	SNNPR	2,071 (37.0)	23 (1.1)	2,048 (98.9)	
	Oromia	1,660 (29.6)	19 (1.1)	1,641 (98.9)	
	Other	403 (7.2)	1 (0.2)	402 (99.8)	
Total		5,600 (100)	59 (1.1)	5,541 (98.9)	

### Prevalence of PTB among homeless individuals

**Figure 1:**
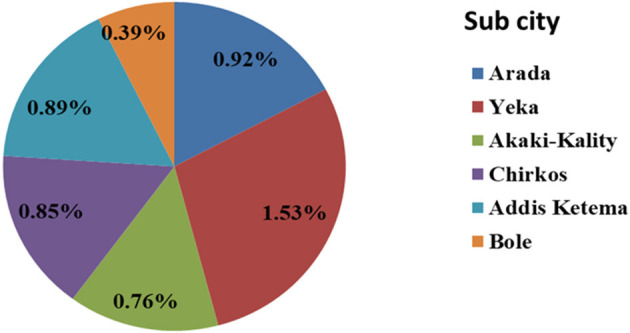
Prevalence of bacteriological confirmed PTB among homeless individuals in sub cities of Addis Ababa, Addis Ababa, Ethiopia, 2021.

GeneXpert and LJ culture were performed for 641 sputa samples of which 59 sputa were positive for PTB in both tests. The prevalence of bacteriological confirmed PTB in homeless individuals was 1.1% (59/5,600) or 10.54 per 1,000 population. The highest (1.53%) prevalence of PTB was recorded in Yeka subcity, while the lowest (0.39%) was in Bole sub city (Figure 1). Co-infection with HIV was observed in 32.2% (19/59) of bacteriologically confirmed TB cases (Table 1). The 582 homeless individuals with symptoms suggestive of TB who were bacteriologically negative were treated with antibiotics for pneumonia for 2 weeks and all were recovered.

### Factors associated with the occurrence of PTB among homeless individuals

All variables significantly associated with the prevalence of bacteriological confirmed PTB in the binary logistic regression analysis remained significant in the multivariable logistic regression analysis except alcohol consumption and drug abuse. Accordingly, being homeless for more than 5 years [AOR = 3.99, 95% CI: 1.31, 11.59], BMI < 18.5 [AOR = 3.20, 95% CI : 1.68, 6.18], smoking cigarette[AOR = 5.71, 95% CI: 1.29, 25.28], living or sleeping in a restricted area with more than five individuals [AOR = 7.82 , 95% CI: 1.02, 60.16], close contact with chronically coughers [AOR = 3.53, 95% CI: 1.14, 10.91], history of imprisonment (AOR = 2.26, 95% CI: 1.08,5.89) and HIV infection [AOR = 2.34 , 95% CI: 1.17, 4.71] were significantly associated with the prevalence of PTB (*P* < 0.05) (Table 2).

**Table 2:** Factors associated with bacteriological confirmed PTB among homeless individuals (*n* = 5,600), Addis Ababa, Ethiopia, 2021.

**Variables/characteristics**		**Frequency (%)**	**AOR (95%CI)**	***P*-value**
Duration being homeless	≥5 years	4,595 (82.1)	**3.99 (1.31, 11.59)**	**0.014**
	< 5 years	1,005 (17.9)	1	
Body mass index	< 18.5	1,580 (28.2)	**3.20 (1.68, 6.09)**	**<** **0.000**
	≥18.5	4,020 (71.8)	1	
Alcohol consumption	Yes	4,351 (77.7)	1.88 (0.76, 4.66)	0.173
	No	1,249 (22.3)	1	
Drug use	Yes	1,837 (32.8)	0.54 (0.26, 1.11)	0.093
	No	3,763 (67.2)	1	
Smoking cigarette	Yes	4,667 (83.3)	**5.71 (1.29, 25.28)**	**0.022**
	No	933 (16.7)	1	
Average number of homeless individuals lived/slept together in one restricted area	Five or more persons	4,601 (82.2)	**7.81 (1.09, 60.16)**	**0.048**
	< 5 persons	999 (17.8)	1	
History of imprisonment	Yes	1,083 (19.3)	**2.26 (1.08, 5.89)**	**0.023**
	No	4,517 (80.7)	1	
Close contact with chronically coughers	Yes	4,589 (81.9)	**3.21 (1.10, 9.37)**	**0.033**
	No	1,011 (18.1)	1	
HIV status	Positive	1,182 (21.1)	**2.34 (1.17, 4.71)**	**0.017**
	Negative	4,418 (78.9)	1	

AOR, Adjusted Odds Ratio. The bold values show statistical significance.

## Discussion

The current cross-sectional study was conducted in Addis Ababa, Ethiopia, to investigate the prevalence of PTB and evaluate associated risk factors in homeless individuals. This study is the first study to be conducted in Addis Ababa, the capital of Ethiopia, where the number of homeless individuals is estimated to be high due to the highest rural-urban migration.

Over 80.3% of the homeless individuals were males, which is consistent with the observation of previous similar studies in Brazil (30) and Ethiopia (9). Furthermore, majority of the homeless individuals in the present study were young and TB was observed in this young group of individuals, which is also in agreement with the reports of earlier studies (31, 32). This observation could be due to the socio-economic crisis of low-income countries in which parents in the urban and rural areas could not satisfy the basic needs and demands of their children that leads to migration of the children to the street for search of their needs. This leads to a significant increase in the number of young homeless individuals and high prevalence of TB in this young group (9). Regarding the gender difference, there are different reasons which contribute to higher percentage of males in homeless individuals in Addis Ababa. One reason sociocultural influence in which restricts the females leave their homes to live on the street. Even though the females face significant socioeconomic problems in their parents' homes, it is not easy for female youths to leave their home and to live on the street. The other contributing factor is that female youths have better chances of employment as the majority of them are absorbed as maids in individuals' homes either in the Ethiopia or abroad in the Middle East countries. In addition, most of the cafeterias, bars, and cafes prefer to recruit female youths than male youths.

According to the result of this study, the prevalence of bacteriological confirmed PTB in the homeless individuals was higher than the national prevalence of TB in the general population of Ethiopia (33). Similarly, several studies (32, 34–36) showed that the prevalence of TB in homeless individuals could be 10 times higher than its prevalence in the general population. For example, earlier studies reported prevalence values, which are similar a prevalence value from Marseille (5), Iran (37) and Japan (34). But other studies reported lower prevalence values than the prevalence recorded in this study from London (38), USA (39), India (40) and even from Ethiopia (9). Comparative evaluation of prevalence values of TB in Ethiopia indicated that the current prevalence was nine times higher than that of the general population of Ethiopia (3, 41) and four times higher than that of the general population of the Addis Ababa City (42) which was 0.67% (43). This could be due to the fact that homeless individuals are neglected section of the population for basic medical care and even have less chance of obtaining basic human needs, such as finding shelter and food, over getting medical care (44). With regard to previous origin of the homeless individuals there was no significant variation in prevalence of bacteriological confirmed PTB. Based on the current residence, majority of the bacteriological confirmed PTB cases were found in the Yeka sub city. This may be due to enrollment of large number of homeless individuals in this sub city.

In consistent with the observation of this study, previous studies conducted on homeless individuals in Colombia (45, 46), Poland (47), USA (48), Rome (49) and South Korea (50) reported higher prevalence of TB in homeless individuals than in the general populations of their respective cities. Similarly, a systematic review and meta-analysis of the prevalence of TB in the homeless individuals recorded a range prevalence values that overlaps with the prevalence value recorded by the present study (51, 52). Such higher prevalence of TB in homeless individuals could linked to risk factors, such as poverty, overcrowding, malnutrition, HIV-AIDS, smoking, alcoholism, and drug abuse (1).

The significant association of the various risk factors with the prevalence of bacteriological confirmed PTB could be explained by overcrowding and delayed diagnosis (53). Low BMI and smoking cigarettes were significantly associated with bacteriological confirmed PTB and this observation agrees with studies conducted in Korea (50) and Rome (54). Previous studies indicated that increase in susceptibility to new infection or reactivation of latent TB infection is associated with malnutrition and smoking (36, 49, 54). Similarly, the association between TB-HIV co-infection has been well documented in the USA (28) and Canada (38) which could also be due to increased susceptibility to new infection and or reactivation of latent TB infection because of immunosuppression by HIV.

### Limitations of the study

A chest X-ray was not performed for screening participants with suspected TB; this may have underestimated the true prevalence of PTB. Extrapulomnary tuberculosis was not included in the study due to time and logistic limitations.

## Conclusion

The prevalence of bacteriological confirmed PTB among homeless individuals in Addis Ababa, Ethiopia was nine times higher than its prevalence in the general population of Ethiopia and four times higher than in the general population of Addis Ababa City. This finding suggests that homeless congregations may be hotspots for TB transmission. Therefore, the TB control program should incorporate homeless individuals in the TB prevention and control program

## Data availability statement

The raw data supporting the conclusions of this article will be made available by the authors, without undue reservation.

## Ethics statement

The studies involving human participants were reviewed and approved by the Addis Ababa University, College of Natural and Computational Sciences Institutional Review Board (IRB) and Addis Ababa City Administration Health Bureau. The patients/participants provided their written informed consent to participate in this study.

## Author contributions

TS participated in the conception, design, acquisition of the data, statistical analysis, interpretation of the data, and drafting the manuscript. SM participated in data acquisition and critical edition of the manuscript. BG and MG participated in the analysis, interpretation of the data, and edition of the manuscript. BP and GA contributed in the design, guiding the data collection, interpretation of the result, and edition of the manuscript. All authors approved the manuscript for publication and agreed to be accountable for all aspects of the work done.
